# Should extragonadal germ cell tumors be included in studies of families with testicular germ cell tumors?

**DOI:** 10.1186/1897-4287-11-1

**Published:** 2013-03-04

**Authors:** Rodrigo Santa Cruz Guindalini, Edite Paulo de Oliveira, Marina Cavalcanto Moroja Silvino, Paulo Marcelo Hoff, Bernardo Garicochea

**Affiliations:** 1 Instituto do Câncer do Estado de São Paulo, Faculdade de Medicina da Universidade de São Paulo, Av. Dr. Arnaldo 251, São Paulo ZIP 01246-000, Brazil; 2 Centro de Oncologia, Hospital Sírio Libanês, São Paulo, Brazil

**Keywords:** Genetic susceptibility, Familial testicular germ cell tumor, Seminoma

## Abstract

**Background:**

Family history is among the few established risk factors for testicular germ cell tumor (TGCT). Approximately 1.4% of newly diagnosed TGCT patients report a positive family history of TGCT. Sons and siblings of TGCT patients have four- to six fold and eight- to tenfold increase in TGCT risk, respectively. In twins of men with TGCT the relative risk of testicular cancer is 37.5 (12.3-115.6). Nevertheless, information about the occurrence of TGCT in relatives of patients with extragonadal germ cell tumor is limited.

**Case report:**

A 24 year-old male patient was diagnosed with a mediastinum tumor and was submitted to image-guided biopsy, which revealed a seminoma. Two months later, his non-identical asymptomatic twin brother was submitted to an elective ultrasound of the testes, which showed a left testicular mass of 4.2 cm. This patient underwent orchiectomy revealing a seminoma of the left testis. There are no other cases of seminoma or other types of cancers reported in first-degree relatives in this family.

**Conclusions:**

Although familial aggregations of TGCT have been well described, to the best of our knowledge, no data concerning the association of gonadal and extragonadal germ cell tumor in relatives has been previously reported. Further investigation on this association is warranted and may help in improving our knowledge of familial pattern inheritance.

## Background

Testicular germ cell tumor (TGCT) is the most common malignancy in males aging 15–35. Known risk factors for TGCT include: cryptorchidism, testicular dysgenesis, infertility, testicular microlithiasis, previously diagnosed TGCT and a family history of the disease [1].

Familial aggregations of TGCT have been well described, suggesting the existence of a hereditary TGCT subset. Approximately 1.4% of newly diagnosed TGCT patients report a positive family history of TGCT [2]. Epidemiological studies have shown that there is an eight to ten fold increase in relative risk of TGCT for brothers of patients and a fourfold increased risk for fathers and sons [3,4]. Moreover, testicular cancer risk was raised in twins of men with testicular cancer (relative risk = 37.5 [12.3-115.6]); risk was greater but not significantly in monozygotic than in dizygotic twins (p = 0.45). The cumulative risk of testicular cancer by the age of 40 years in men whose monozygotic twins had testicular cancer was 14% (4–46) [5]. A previous study using segregation analysis of testicular cancer favored an autosomal recessive model of inheritance [6] and, although no high-penetrance cancer susceptibility gene has been mapped yet, linkage analyses have identified several genomic regions of modest interest [7]. Y chromosome *gr/gr* deletion and *PDE11A* gene mutations have been suggested to modify the risk of familial testicular germ cell tumor (FTGCT) [8,9]. Phenotypic aspects of FTGCT include 2 cases of TGCT per family, mean age at diagnosis about 2.5 years younger than that observed for sporadic TGCT and greater prevalence of bilateral cancer [10].

The establishment of a universal screening program using testicular palpation (clinical or patient self-examination) or serum biomarker for healthy young patients lacks clinical evidence [11]. And, although some risk factors for TGCT are well known, effectiveness of high-risk patients screening is controversial. Recent systematic review did not detect published randomized clinical trials comparing screening versus no screening for testicular cancer [12]. On the basis of uncertain benefits and some likelihood of harms, the US Preventive Services Task Force and the American Academy of Family Physicians discourage screening. But others, such as the American Cancer Society and the European Association of Urology, recommend different approaches: testicular examination to be part of a periodic cancer-related checkup [13] and self-testicular examination for individuals with clinical risk factors [14], respectively.

Extragonadal germ cell tumors account for fewer than 10% of all germ cell malignancies. All reports of family inheritance focused exclusively in isolated germ cell tumors of testis [4,6,7] and the estimation of the contribution of extragonadal germ cell tumor in the familial setting is not known. Therefore, the omission of extragonadal tumors in the published reports may have contributed to the underestimation of the importance of the hereditary factor of germ cell tumors, especially when there is a presence of an affected twin. Here we present a report of simultaneous occurrence of seminomas in dizygotic twins: one in the testicle and the other in the mediastinum.

## Case report

A 24 year-old male patient was first diagnosed with a mediastinal mass. Other lesions were not found elsewhere in the body, including the testicles. A computed tomography-guided transthoracic biopsy led to the diagnosis of primary mediastinal seminoma. Two months after, his non-identical asymptomatic twin brother was then submitted to an elective ultrasound of the testes, which showed a left testicular mass of 4.2 cm. After excluding extragonadal disease, this patient underwent orchiectomy that revealed a pure seminoma of the left testis with rete testis invasion. There are no other cases of seminoma or other types of cancers reported in first-degree relatives of this family. Initially, the first patient received 4 cycles of bleomycin, cisplatin, and etoposide (BEP) and is now following a residual mediastinal mass with serial images. His brother has no evidence of disease after 1 cycle of carboplatin AUC 7 mg/mL.

## Discussion

Although mediastinal germ cell neoplasms account for only 2% to 5% of all germinal tumors, they constitute up to 50% to 70% of all extragonadal tumors. When this mediastinal tumor is identified, it is mandatory to exclude TGCT. Daugaard et al. reported that, while carcinoma in situ testis (CIST) was diagnosed in 42% of germ cell primary tumors of retroperitoneum, indicating that these tumors are probably not truly extragonadal, none of the patients with tumors in mediastinum who underwent biopsy had CIST [15]. Accordingly, the vast majority of patients with mediastinal tumor will not develop testicular tumors and, as a consequence, will not be analyzed in epidemiological studies using population databases. In this sense, although the familial association of gonadal and extragonadal germ cell tumors has not yet been established, based on the present case report it is possible to question if the exclusion of extragonadal germ cell tumors from FTGCT studies may exert a negative influence on the advances of the knowledge regarding the underlying common genetic mechanisms of germ cell tumors.

Furthermore, it is important to note that the decision of performing the investigation on the asymptomatic brother was closely guided by the information provided in previous studies of FTGCT [1]. This initiative permitted an early tumor diagnosis and, consequently, greater chances of successful treatment without an unnecessary exposure to the acute and late toxicities of BEP. In this perspective, we would like to propose the discussion of whether we should start including extragonadal germ cell tumors in studies of families with testicular germ cell tumors.

Nevertheless, further investigations in other clinical and population based studies are necessary and may improve our understanding of familial pattern of inheritance. We believe that future research on the association of testicular and extragonadal germ cell tumors in the familial setting may provide new insights and discoveries in the field and, hopefully, help on the design of strategies for prevention, screening and follow-up of families with germ cell tumors, especially when there is a presence of an affected twin.

## Consent

Written informed consent was obtained from the patients for publication of this Case report. A copy of the written consent is available for review by the Editor-in-Chief of this journal.

## Abbreviations

BEP: Bleomycin, cisplatin, and etoposide; CIST: Carcinoma in situ testis; FTGCT: Familial testicular germ cell tumor; TGCT: Testicular germ cell tumor.

## Competing interests

The authors declare that they have no competing interests.

## Authors’ contributions

RSCG made substantial contributions to conception and design, and was involved in drafting the manuscript. MCMS and EPO performed the acquisition of data and the literature review. PMH conducted and coordinated the case. BG conceived the case report, and participated in its design and helped to draft the manuscript. All authors read and approved the final manuscript.
